# Supplementary material for: Spontaneous traveling waves naturally emerge from horizontal fiber time delays and travel through locally asynchronous-irregular states
Source: Nat Commun. 2021 Oct 18;12:6057. doi: 10.1038/s41467-021-26175-1 (PMC8523565; doi:10.1038/s41467-021-26175-1)
Supplement: Supplementary file 1 — Supplementary Information [file 41467_2021_26175_MOESM1_ESM.pdf]

Supplementary Information

Supplementary Figure 1

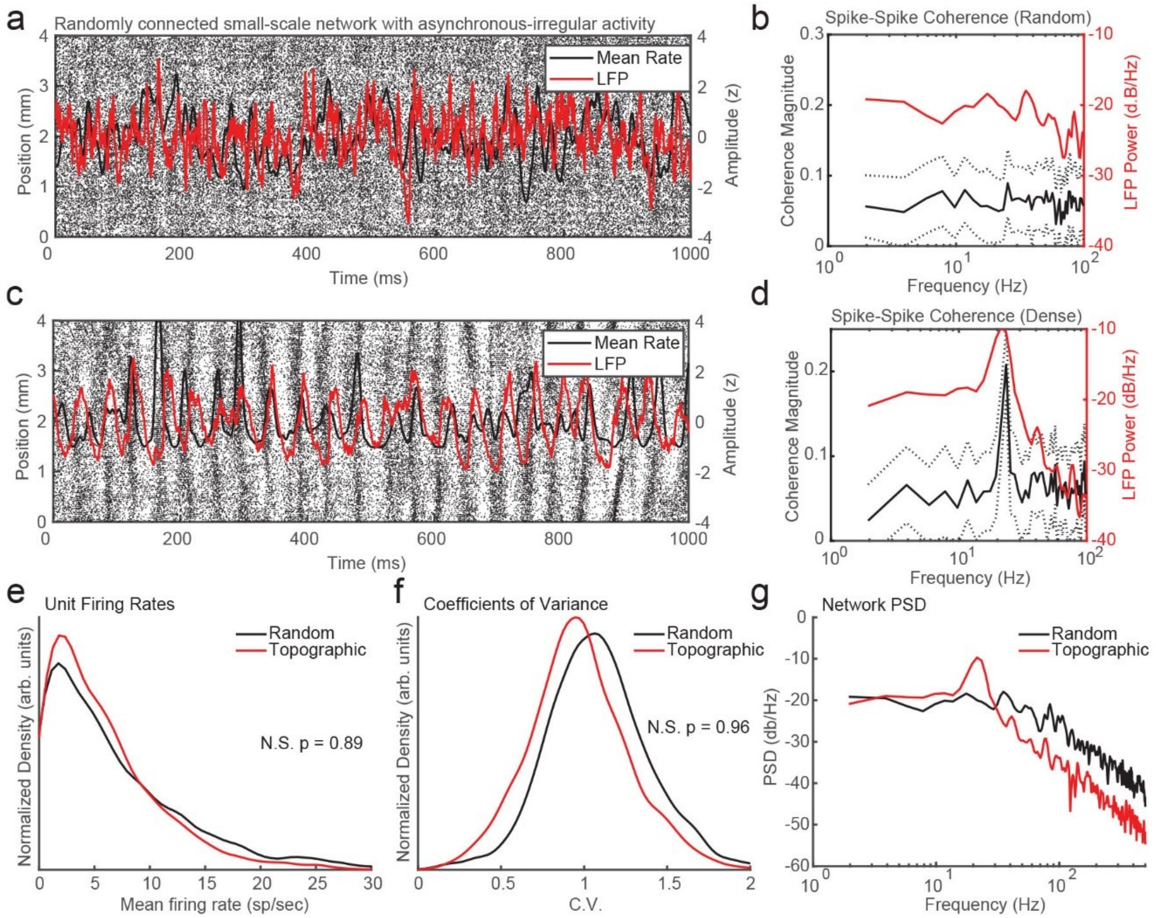

Figure S1. Dense spiking waves induce pairwise correlation in a small-scale spiking network. (a) Spike rasters from 10,000 neurons in a small-scale network simulation with random connections and no delays producing asynchronous-irregular spiking dynamics. The mean firing rate and LFP for a single 100 neuron LFP pool is plotted in black and red respectively. (b) Pairwise spike coherence (black line) and power spectral density (red line) for LFP pools in (a). Dotted lines denote the 95% confidence interval of the mean. (c) Spike rasters as in (a), but with topographic connections and transmission delays (0.2 m/s). (d) Coherence and PSD as in (b) but for the network shown in (c). Dense spike participation in waves generates strong coherence at the dominant frequency of fluctuations in the network. (e) There was no difference in the distribution of unit mean firing rates between the random (black) and topographic (red) small-scale networks (N = 5000 units; p = 0.89; two-sided Kolmogorov–Smirnov test). (f) There was no significant difference in the distribution of unit C.Vs between the random and topographic small-scale

networks (N = 3319 units; p = 0.96; two-sided Kolmogorov–Smirnov test). (g) Comparison of the PSD between the random and topographic small-scale networks.

Supplementary Figure 2

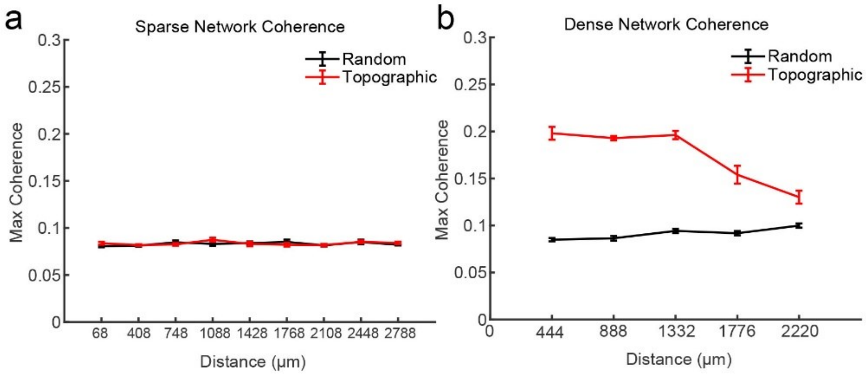

Figure S2. Distance dependence of pairwise spike coherence. (a) The maximum pairwise spike coherence calculated between neuron pools at various distances in the random (black) and topographic (red) large-scale networks in Figure 2. There was no change in spike coherence in either network at any distance. (b) Same as (a), but for the small-scale networks in Figure S1. There was a negative correlation with maximum spike coherence and distance in the small-scale topographic model (N = 10 resamples, error bars indicate S. E. M.; Pearson's r = -0.72).

Supplementary Figure 3

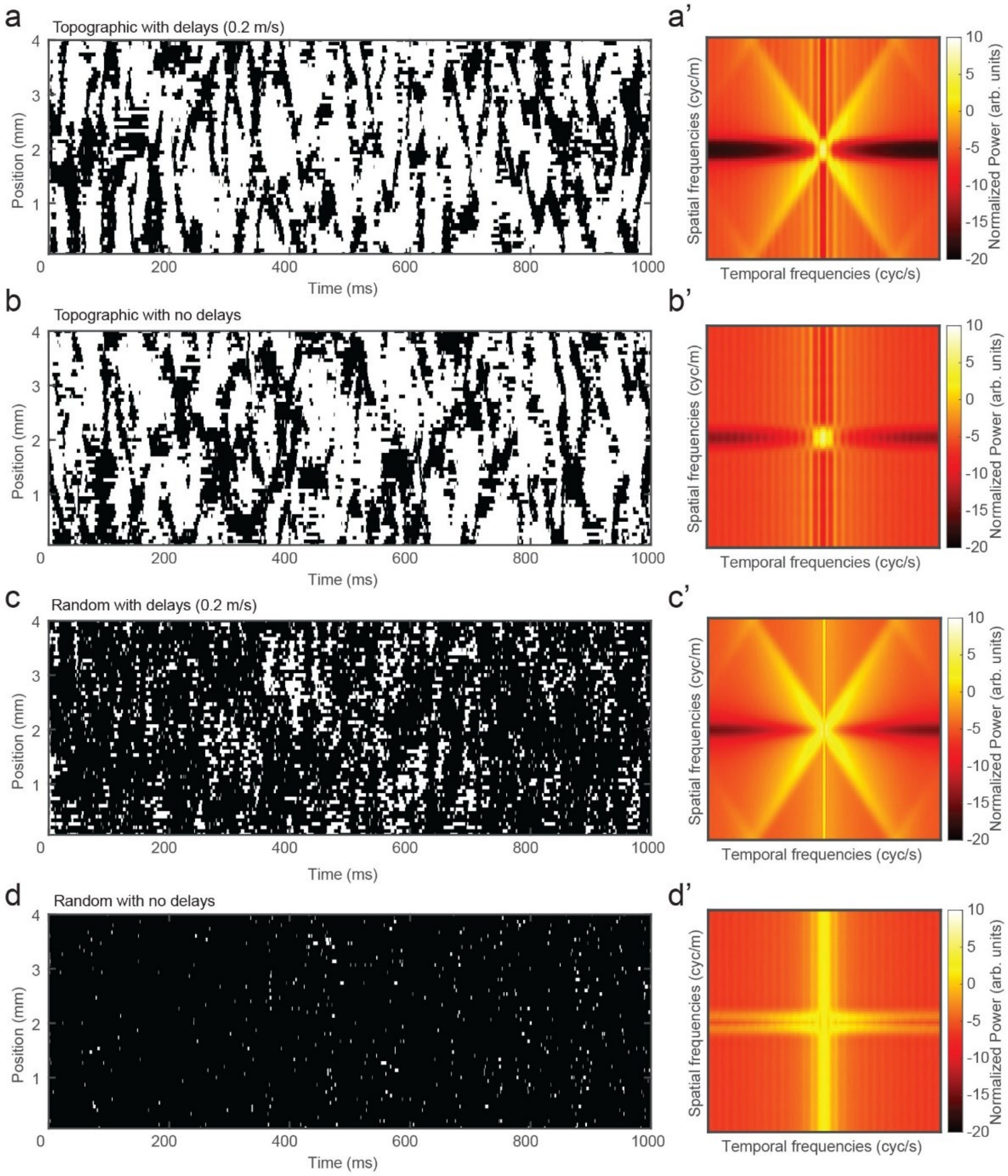

Figure S3. Topographic connections and distance-dependent delays combined are necessary to generate traveling waves. (a) Significant (white) and non-significant (black) wavelength values for each position in a linear slice through a large-scale 2D network simulation with topographic connections and no delays. (a') 2-D (space-time) FFT shows a concentration of spectral power corresponding to waves traveling at the velocity corresponding to propagation speeds (0.2 m/s). (b, b') Same as in (a), but for a network with topographic connectivity and no delays. Topographic connectivity is sufficient to generate significant spatially organized wavelengths. However, without delays, the spectral power does not concentrate along a joint spatial and temporal frequency band consistent with traveling waves. (c, c') Wavelengths and spatiotemporal FFT for

a randomly connected network with delays. With random connectivity the network lacks strong spatial organization while delays are sufficient for the spatiotemporal flow of activity. (d, d'). Wavelengths and spatiotemporal FFT for a randomly connected network without delays. There is no spatial or temporal structure in this network suggestive of any wave activity.

Supplementary Figure 4

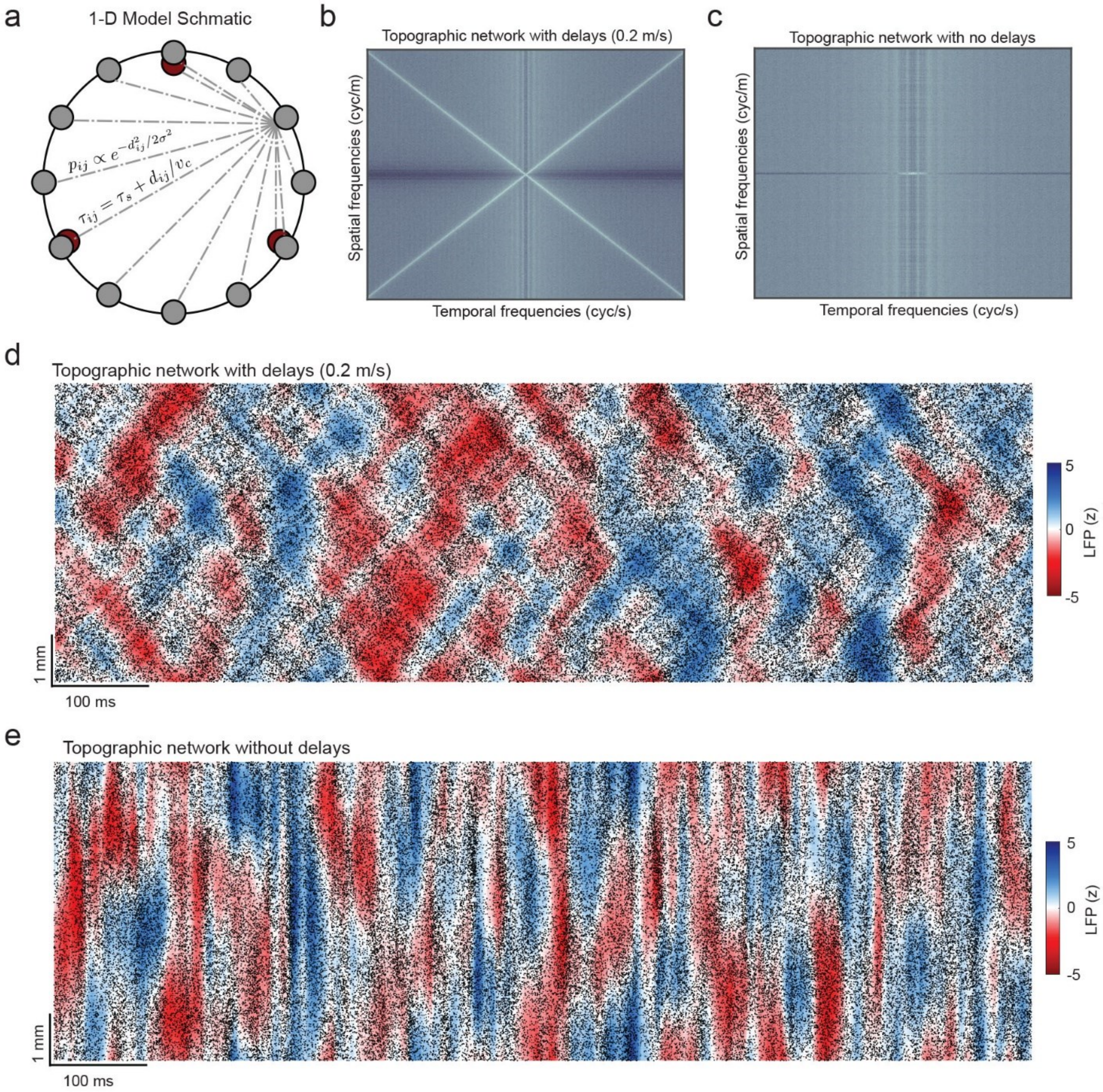

Figure S4. Delays are necessary for robust traveling waves in 1-D spiking network model. (a) Schematic of 1-D network model. 450,000 neurons were arranged on a ring with topographic connection probabilities and distance dependent delays. (b) 2-D FFT of the spatial (y-axis) and temporal (x-axis) frequencies of activity in the topographic network. The clear spectral line is consistent with waves traveling at 0.2 m/s. (c) No spectral line appears in a similar topographic 1D network without delays. (d) Spike rasters and LFP amplitude (pseudocolor) for the topographic network displays waves moving across space over time in the 1-D topographically connected network with delays. (e) Same as (d), but for the 1-D topographic network without

delays. LFP fluctuations do not travel as waves but rather occur synchronously across regions of the network.

Supplementary Figure 5

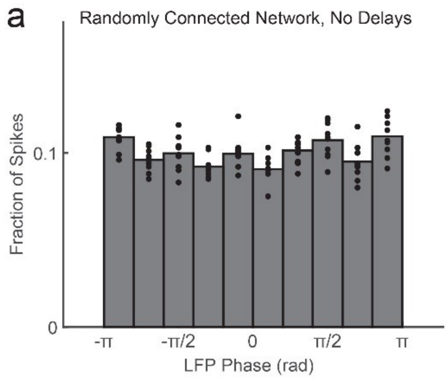

Figure S5. Randomly-connected spiking network model has weak spike-LFP phase coupling. (a) Histogram showing the fraction of spikes that occurred during each phase of the LFP in the randomly connected network shown in Figure 2a (N = 10 resamples; circular-resultant = 0.03).

Parameter		Random Network	Topographic Network	Dense Network	1-D Network
N	# of neurons	1,012,500	1,012,500	12,500	450,000
N_e	# of excitatory neurons	810,000	810,000	10,000	360,000
N_i	# of inhibitory neurons	202,500	202,500	2,500	90,000
K	out-synapses per neuron	3,000	3,000	100	3,000
L	network side length	6 mm	6 mm	4 mm	5.66 mm
C_m	membrane capacitance	200 pF	200 pF	200 pF	200 pF
G_L	leak conductance	10 nS	10 nS	10 nS	10 nS
E_L	resting membrane potential	-65 mV	-65 mV	-65 mV	-65 mV
τ_e	excitatory synaptic time constant	5 ms	5 ms	5 ms	5 ms
τ_i	inhibitory synaptic time constant	5 ms	5 ms	5 ms	5 ms
E_e	excitatory reversal potential	0 mV	0 mV	0 mV	0 mV
E_i	inhibitory reversal potential	-80 mV	-80 mV	-80 mV	-80 mV
V_t	threshold potential	-50 mV	-50 mV	-50 mV	-50 mV
V_r	reset potential	-70 mV	-70 mV	-70 mV	-70 mV
G_e	excitatory synaptic weight	1 nS	1 nS	4 nS	1 nS
G_i	inhibitory synaptic weight	10 nS	10 nS	490 nS	10 nS
τ_r	refractory period	5 ms	5 ms	5 ms	5 ms
σ	standard deviation of Gaussian	N/A	400 μm	400 μm	566 μm
v_c	axonal conduction speed	N/A	0.2 m/s	0.2 m/s	0.2 m/s
τ_s	synaptic vesicle delay	300 μs	300 μs	300 μs	300 μs

Supplemental Table S1. Parameters used in the simulations of the random network, topographic network, dense network, and 1-D network.

Parameter	0.5 mm network	1 mm network	2 mm network	3 mm network	4 mm network
N # of neurons	8,000	28,125	112,500	253,125	450,000
K out-synapses per neuron	100	375	1500	3,000	3,000
G_e excitatory synaptic weight	0.1-5 nS	0.1-5 nS	0.1-5 nS	0.1-5 nS	0.1-5 nS
G_i inhibitory synaptic weight	1-50 nS	1-50 nS	1-50 nS	1-50 nS	1-50 nS
L network side length	0.533 mm	1 mm	2 mm	3 mm	4 mm

Supplemental Table S2. Parameters used in scans across 2-D network sizes. N scales with L to maintain a constant neuronal density. K scales with N until the point L exceeds the area of the Gaussian that determines the probability of neuronal connections. The corresponding values of all parameters not listed here are found in Table S1 under the topographic network.
