# Supplementary material for: Spontaneous traveling waves naturally emerge from horizontal fiber time delays and travel through locally asynchronous-irregular states
Source: Nat Commun. 2021 Oct 18;12:6057. doi: 10.1038/s41467-021-26175-1 (PMC8523565; doi:10.1038/s41467-021-26175-1)
Supplement: Supplementary file 3 — Description of Additional Supplementary Files [file 41467_2021_26175_MOESM3_ESM.pdf]

Description of Additional Supplementary Files

File Name: Supplementary Movie 1
Description: Traveling waves were present in waking spontaneous activity in behaving marmosets. 100 ms of spontaneous activity recorded from a Utah multi-electrode recording array chronically implanted in area MT of a common marmoset. The marmoset was holding fixation awaiting the appearance of a faint visual target. The spatial extent of the recording covered 3.2 x 3.2 mm.

File Name: Supplementary Movie 2
Description: The randomly connected spiking network model produced noisy homogeneous LFP fluctuations across the spatial extent of the network. 200 ms of spontaneous activity in a randomly connected large-scale spiking network model as described in Figure 2. Each pixel is the amplitude of the LFP calculated from synaptic activity in each 10x10 neuron pool.

File Name: Supplementary Movie 3
Description: The topographically connected spiking network model produced heterogeneous LFP fluctuations that travel as waves across the spatial extent of the network. 200 ms of spontaneous activity in a topographically connected large-scale spiking network model described as in Figure 2. The distance-dependent connections were formed from a 2D Gaussian with sigma = 400 µm and the conduction velocity was 0.2 m/s.

File Name: Supplementary Movie 4
Description: Wavelengths are shorter in a network with a smaller Gaussian sigma. 200 ms of spontaneous activity in a topographically connected large-scale spiking network with a Gaussian sigma of 200 µm and a conduction velocity of 0.2 m/s.

File Name: Supplementary Movie 5
Description: Wavelengths are longer in a network with a larger Gaussian sigma. 200 ms of spontaneous activity in a topographically connected large-scale spiking network with a Gaussian sigma of 600 µm and a conduction velocity of 0.2 m/s.
