# Supplementary material for: Spontaneous traveling waves naturally emerge from horizontal fiber time delays and travel through locally asynchronous-irregular states
Source: Nat Commun. 2021 Oct 18;12:6057. doi: 10.1038/s41467-021-26175-1 (PMC8523565; doi:10.1038/s41467-021-26175-1)
Supplement: Supplementary file 9 — Reporting Summary [file 41467_2021_26175_MOESM9_ESM.pdf]

nature research

Corresponding author(s): Zachary W. Davis, John Reynolds, Lyle Muller

Last updated by author(s): 08/16/2021

Reporting Summary

Nature Research wishes to improve the reproducibility of the work that we publish. This form provides structure for consistency and transparency in reporting. For further information on Nature Research policies, see our Editorial Policies and the Editorial Policy Checklist.

Statistics

For all statistical analyses, confirm that the following items are present in the figure legend, table legend, main text, or Methods section.

n/a	Confirmed	
☐	☒	The exact sample size (*n*) for each experimental group/condition, given as a discrete number and unit of measurement
☐	☒	A statement on whether measurements were taken from distinct samples or whether the same sample was measured repeatedly
☐	☒	The statistical test(s) used AND whether they are one- or two-sided<br>*Only common tests should be described solely by name; describe more complex techniques in the Methods section.*
☒	☐	A description of all covariates tested
☒	☐	A description of any assumptions or corrections, such as tests of normality and adjustment for multiple comparisons
☐	☒	A full description of the statistical parameters including central tendency (e.g. means) or other basic estimates (e.g. regression coefficient) AND variation (e.g. standard deviation) or associated estimates of uncertainty (e.g. confidence intervals)
☐	☒	For null hypothesis testing, the test statistic (e.g. *F*, *t*, *r*) with confidence intervals, effect sizes, degrees of freedom and *P* value noted<br>*Give P values as exact values whenever suitable.*
☒	☐	For Bayesian analysis, information on the choice of priors and Markov chain Monte Carlo settings
☒	☐	For hierarchical and complex designs, identification of the appropriate level for tests and full reporting of outcomes
☐	☒	Estimates of effect sizes (e.g. Cohen's *d*, Pearson's *r*), indicating how they were calculated

Our web collection on statistics for biologists contains articles on many of the points above.

Software and code

Policy information about availability of computer code

Data collection	Electrophysiology data were collected using Intan RHD 2000 Interface version 1.4.2. Behavioral data were collected using MonkeyLogic version 1.2.0 running in Matlab version 2020a. Spike sorting was performed using MClust (ver. 4.3.02; A. David Redish, University of Minnesota). Spiking network simulations were generated from our custom, open-source software NETSIM (v0.1 available at https://github.com/mullerlab/NETSIM) and was run on the San Diego Supercomputer Center (SDSC) COMET supercomputer cluster.
Data analysis	All data analyses were performed using custom code running in MATLAB R2020a. The code is available at https://github.com/mullerlab/davis2021ncomms.

For manuscripts utilizing custom algorithms or software that are central to the research but not yet described in published literature, software must be made available to editors and reviewers. We strongly encourage code deposition in a community repository (e.g. GitHub). See the Nature Research guidelines for submitting code & software for further information.

Data

Policy information about availability of data

All manuscripts must include a data availability statement. This statement should provide the following information, where applicable:

- Accession codes, unique identifiers, or web links for publicly available datasets
- A list of figures that have associated raw data
- A description of any restrictions on data availability

The data that support the findings in the main figures of this study are available at https://github.com/mullerlab/davis2021ncomms.

Field-specific reporting

Please select the one below that is the best fit for your research. If you are not sure, read the appropriate sections before making your selection.

☒ Life sciences ☐ Behavioural & social sciences ☐ Ecological, evolutionary & environmental sciences

For a reference copy of the document with all sections, see nature.com/documents/nr-reporting-summary-flat.pdf

Life sciences study design

All studies must disclose on these points even when the disclosure is negative.

Sample size	Two marmoset monkeys were used for this study. No sample size calculation was performed. The number used was chosen to comply with ethical considerations to reduce the number of animals used in experimental research. The results were collected from the first monkey and replicated in a second as is the standard practice in primate neuroscience research.
Data exclusions	For analyses of spontaneous activity, data was only included if it was during a period of at least 300 ms of fixation. For the network simulations the initial 200 ms of the simulation time was excluded to remove and initialization confounds from the steady-state activity.
Replication	Experimental results from the first monkey were replicated in a second monkey. The results of the computational simulations were replicated multiple times on separate computers across institutions.
Randomization	Randomization is not relevant for this study as there are no experimental groups.
Blinding	Blinding was not relevant to this study as no experimental group was defined.

Reporting for specific materials, systems and methods

We require information from authors about some types of materials, experimental systems and methods used in many studies. Here, indicate whether each material, system or method listed is relevant to your study. If you are not sure if a list item applies to your research, read the appropriate section before selecting a response.

Materials & experimental systems

n/a	Involved in the study	
☒	☐	Antibodies
☒	☐	Eukaryotic cell lines
☒	☐	Palaeontology and archaeology
☐	☒	Animals and other organisms
☒	☐	Human research participants
☒	☐	Clinical data
☒	☐	Dual use research of concern

Methods

n/a	Involved in the study	
☒	☐	ChIP-seq
☒	☐	Flow cytometry
☒	☐	MRI-based neuroimaging

Animals and other organisms

Policy information about studies involving animals; ARRIVE guidelines recommended for reporting animal research

Laboratory animals	Two adult marmosets (Callithrix jacchus) were used in this study (male, 4 years of age; female, 3 years of age).
Wild animals	The study did not involve wild animals.
Field-collected samples	The study did not involve samples collected from the field.
Ethics oversight	All experimental methods were approved by the Institutional Animal Care and Use Committee (IACUC) of the Salk Institute for Biological Studies and conformed with NIH guidelines.

Note that full information on the approval of the study protocol must also be provided in the manuscript.
